# Implicit Recognition Based on Lateralized Perceptual Fluency 

**DOI:** 10.3390/brainsci2010022

**Published:** 2012-02-06

**Authors:** Iliana M. Vargas, Joel L. Voss, Ken A. Paller

**Affiliations:** 1 Department of Psychology, Northwestern University, Evanston, IL 60208, USA; E-Mail: iliana.m.vargas@u.northwestern.edu; 2 Department of Medical Social Sciences, Northwestern University Feinberg School of Medicine, Chicago, IL 60611, USA; E-Mail: joel-voss@northwestern.edu

**Keywords:** implicit memory, recognition, perceptual priming

## Abstract

In some circumstances, accurate recognition of repeated images in an explicit memory test is driven by implicit memory. We propose that this “implicit recognition” results from perceptual fluency that influences responding without awareness of memory retrieval. Here we examined whether recognition would vary if images appeared in the same or different visual hemifield during learning and testing. Kaleidoscope images were briefly presented left or right of fixation during divided-attention encoding. Presentation in the same visual hemifield at test produced higher recognition accuracy than presentation in the opposite visual hemifield, but only for guess responses. These correct guesses likely reflect a contribution from implicit recognition, given that when the stimulated visual hemifield was the same at study and test, recognition accuracy was higher for guess responses than for responses with any level of confidence. The dramatic difference in guessing accuracy as a function of lateralized perceptual overlap between study and test suggests that implicit recognition arises from memory storage in visual cortical networks that mediate repetition-induced fluency increments.

## 1. Introduction

Recognition is the ability to correctly identify a previously experienced stimulus as such and is normally considered a hallmark of explicit memory. *Explicit memory* refers to the ability to consciously recognize or recall previously experienced information. In contrast, *implicit memory* refers to various other instances when stored information influences behavior without awareness of memory retrieval. For example, *perceptual implicit memory* for visual stimulus features can be shown by faster and more accurate responses in a perceptual priming test and is believed to occur through repetition-induced fluency of perceptual processing within visual cortex [1,2]. Specific neural mechanisms that might underlie repetition-induced fluency are currently under active debate [3]. A related controversy concerns the extent to which this repetition-induced fluency contributes to explicit memory as commonly assessed in a recognition test.

Jacoby’s [4] process-dissociation methodology presented an alternative to contrasting implicit memory tests with explicit memory tests. He argued that a combination of automatic and intentional processes contribute to memory performance, and that memory tests do not provide pure measures of one or the other type of process. Applied to results from recognition testing, the process-dissociation methodology represents an attempt to separate the contribution of these different processes. This tactic thus acknowledges the idea that an explicit memory test can engage an automatic component of performance (perhaps related to implicit memory). Evidence consistent with the idea that recognition responses can be driven by implicit-memory processes akin to those thought to support perceptual priming can be seen in various applications of the process-dissociation methodology as well as in a variety of other experimental protocols (e.g., [5,6,7,8]). Influences of relatively automatic processing (e.g., perceptual fluency) on recognition performance may be greatest when semantic elaboration is minimal [9] and when explicit memory is weak [10,11]. Indeed, a direct influence of manipulations of perceptual fluency has been observed in various studies of recognition (e.g., [12,13,14]). Also, superior memory for stimuli studied and tested in the same *versus* a different modality in both explicit and implicit memory tests has been attributed to similar processes operating in both types of test [15]. On the other hand, other results suggest that perceptual fluency does not contribute to recognition decisions (e.g., [16,17]). Most dramatically, some amnesic patients can achieve only chance levels of recognition accuracy but also exhibit normal implicit memory for the same stimuli on various priming tests [18,19]. Given that implicit memory was operative at normal levels in these amnesic patients, a reasonable conclusion is that implicit memory does not contribute to recognition, because if it did then recognition performance should have been above chance.

The controversy about whether implicit memory can or cannot support recognition performance seems tied to settling on a general answer to this question. As opposed to an all-or-none orientation, however, the question might best be framed by asking whether certain circumstances promote *accurate recognition driven by perceptual fluency without awareness of retrieval*, which we have termed *implicit recognition*. In other words, to settle this controversy it may prove helpful to identify task and stimulus parameters that promote implicit recognition, which would open the door to considering the degree to which implicit recognition may be operative in various circumstances.

In a recognition test, implicit recognition may occur on an unknown subset of trials. In order to obtain convincing evidence demonstrating implicit recognition, it must occur reliably on a large number of trials. In a series of studies, we therefore used stimulus and task parameters that we thought would maximize implicit recognition. We also made use of confidence judgments, such that a lack of confidence (*i.e.*, a guess) would be one way to indicate a lack of awareness of retrieval. Although these metamemory judgments might be subject to various biases and may not always be veridical, consistently different findings for guesses and confident recognition responses can be highly informative.

We obtained our initial evidence for implicit recognition using forced-choice tests of recognition memory for previously seen kaleidoscope images [20,21,22]. These stimuli tend to provoke very little conceptual processing, though some color naming may occur during learning. However, the same colors were used in to-be-learned stimuli and in foil stimuli presented during recognition testing, such that memory for color names was seldom diagnostic during a test. Successful recognition was thus heavily based on visual features.

A key manipulation that produced results implicating implicit recognition involved manipulating attention during encoding. This experimental manipulation functions as an assay for explicit memory, given that full attention typically produces better explicit memory than divided attention [23,24,25]. When kaleidoscope images were studied under divided attention, they were recognized more accurately than when studied under full attention [20,21]. This counterintuitive outcome (contrary to expectations if explicit memory solely supported recognition) was observed when recognition decisions were made rapidly after test kaleidoscope images appeared, but not when subjects were instructed to wait several seconds longer and to consider their choices more thoroughly [20]. At test, implicit recognition was demonstrated using two-alternative forced-choice testing procedures with perceptually similar foils. Under such conditions, confident responses were less accurate than guesses, which would also seem to epitomize recognition without awareness of retrieval [20,21]. In contrast, when the test required a yes/no judgment or a forced choice with non-corresponding foils, recognition was no longer superior after encoding with divided attention than with full attention [20].

Although these studies identified some of the boundary conditions for implicit recognition, they did not address all relevant issues. Other critical features of the procedure were brought to the forefront when a failure to replicate was reported by Jeneson and colleagues [26]. This report led us to consider our procedures in a new light; another critical factor influencing the results might be that our subjects were exceedingly willing to guess, whereas subjects in the Jeneson study labeled fewer of their responses as guesses. Accordingly, we conducted a within-subjects experiment to specifically manipulate subjects’ orientation to produce guesses. When guessing was encouraged during testing, guesses were more prevalent and more accurate than when confident responses were encouraged [22]. It appears that subjects were more likely to use perceptual fluency when they were comfortable with guessing and not striving for confident retrieval experiences. Likewise, the extreme difficulty of the test and the generally poor ability to remember stimuli exactly might also be relevant factors that encourage implicit recognition.

Physiological findings also provided very convincing support for our demonstration of implicit recognition. In one experiment with the same basic design as described above, neural correlates of correct guessing [21] resembled those linked with perceptual priming in other experiments [27,28,29]. Specifically, event-related potentials (ERPs) during implicit recognition showed greater negativity for correct guesses compared to new stimuli at approximately 200–400 ms after stimulus onset. Similar ERPs were elicited by repeated kaleidoscope images in a priming test requiring a three-choice decision about the number of colors in each image, and perceptual priming was concurrently demonstrated by faster and more accurate decisions for repeated than for new stimuli [27]. In contrast, ERPs associated with any level of explicit memory confidence (including low-confidence familiarity responses) were distinct, taking the form of positive deflections that occurred primarily with later onset. The finding that the same neural signal seems to occur with implicit recognition and with perceptual priming, and that this signal is distinct from signals of explicit memory, provides an independent link between these implicit memory expressions.

We propose that implicit recognition in these experiments resulted from perceptual fluency that influenced responding without awareness of memory retrieval. Although the extant data are insufficient for determining the precise neural sources of this fluency, it likely arises from plasticity in a subset of the neurons in visual cortex regions responsible for object perception. Neurophysiological memory theories propose that memory processing is dependent on synaptic changes occurring in areas where information was originally processed [30]. Therefore, memory traces for visual information should be found along the same pathways where item-specific information processing occurred, conforming to the contralateral organization of early visual processing [31]. For example, evidence for the contralateral organization of visual memories was obtained in an experiment reported by Gratton and colleagues [32]. Recognition for line patterns was greater when there was a match in hemifield presentation during study and test. Presumably, when a stimulus was shown to only one visual hemifield, a stronger memory trace was formed in contralateral visual cortex than in ipsilateral visual cortex. A match between the memory trace and the sensory representation at test thus increased the chances the stimulus would be recognized. Additionally, ERPs recorded when test stimuli were presented centrally had lateralized distributions based on the hemifield of study. Specifically, there was greater negativity for the hemisphere contralateral to the visual hemifield where items were originally presented, consistent with the notion of lateralized neural storage for these stimuli. 

One prediction from the above considerations is that implicit recognition for kaleidoscope images should vary as a function of whether learning and testing concern stimuli in the same or different visual hemifields. Accordingly, we extended the procedures used in our previous experiments and presented lateralized kaleidoscope images. We used divided-attention procedures during learning and tested recognition using a two-alternative forced-choice recognition test with a highly similar foil on each trial. At learning, some kaleidoscope images were presented in the left visual hemifield and some in the right visual hemifield. At test, kaleidoscope images were presented on the same side as learning or on the opposite side. Thus, we were able to determine the extent to which accurate implicit recognition depends upon the lateralized organization of visual memory storage.

## 2. Methods

Visual stimuli included 344 kaleidoscope images (henceforth referred to as kaleidoscopes). Kaleidoscopes were created by overlaying three opaque hexagons of different colors and performing three rounds of side bisection and random deflection on each. A high degree of similarity between the members of 160 pairs was obtained by using the same three colors and deflecting each matching-color hexagon at similar random angles (with <10° difference). These 160 pairs included 144 pairs of study items and their corresponding foils and 16 pairs of kaleidoscopes that appeared only during the recognition test. In addition to these 320 kaleidoscopes, another 24 appeared only during learning in order to guard against primacy and recency effects.

Memory was assessed in 24 individuals (ages 18–22; 14 female) using 12 study-test blocks. The experiment began with a period of fixation training using a flicker-display technique that helps participants reduce the number of eye movements during visual fixation [33]. Additionally, participants practiced the divided-attention task that they would have to perform later. During this practice, participants maintained fixation on a central gray cross with two green squares on either side, and made one-back odd/even judgments for a series of spoken digits. They were instructed to indicate with a button press whether the digit for the previous trial was odd or even.

Each study session included 50 trials in which a set of 12 novel kaleidoscopes were repeatedly presented for learning. The 50 trials included a primacy buffer, 4 presentations of each to-be-learned kaleidoscope, and a recency buffer. In each set of 12 trials after the primacy buffer, all 12 kaleidoscopes appeared in random order with the constraint that the same kaleidoscope could never appear twice in succession. Each kaleidoscope appeared on a black background either on the left or the right of a central gray fixation cross while a gray circle appeared on the opposite side. The average kaleidoscope size was 7.6 cm in diameter (4.7° visual angle) with an average eccentricity of 3.3° visual angle (measured from fixation to center of kaleidoscope). The distance between the display and the subject was 92 cm. The gray circles were included so that some concurrent visual input was sent to each hemisphere. Each presentation was for 250 ms followed by a 1750-ms interstimulus interval. Subjects were instructed to memorize the kaleidoscopes while maintaining central fixation. Half of the kaleidoscopes consistently appeared on the left and the other half on the right. Encoding occurred in conjunction with the same divided-attention task practiced earlier. A spoken digit was presented beginning with the onset of each kaleidoscope. For each study trial (except the first), subjects pressed a button to indicate whether the digit spoken on the previous trial was odd or even. 

A recognition test followed each study session after a 45-s delay, during which subjects performed an arithmetic task and were reminded of the test instructions. The experimenter repeatedly stressed that the test would be very difficult and that participants should not worry about always making correct responses. They were told to try their best and that due to the difficulty of the task it was perfectly fine to guess when necessary. For each of 12 trials, one of the studied kaleidoscopes and its corresponding visually similar foil were presented on opposite sides of a gray central fixation cross for 1000 ms followed by a 3500-ms interstimulus interval with a gray fixation cross that turned red for the final 1000 ms of the interstimulus interval prior to the next trial. Subjects were instructed to make a response after the two kaleidoscopes disappeared and before the fixation cross turned red. Subjects were told to fixate the central cross throughout the test. Kaleidoscopes were presented at exactly the same spatial locations as during study. Half of the studied kaleidoscopes were presented on the same side as during learning and the other half were presented on the opposite side. Another one or two trials that occurred at random times during each test consisted of two new kaleidoscopes that were highly similar. There were a total of 16 of these *new-new* trials for each subject, and they were included in order to reveal the baseline proportion of guess responses produced without any prior stimulus presentation. The addition of these trials might have also increased the overall level of perceived difficulty of the test, given that subjects were not told about new-new trials. Subjects were instructed to select the old kaleidoscope using 4 response choices: 1 = left kaleidoscope with any level of confidence; 2 = left kaleidoscope as a guess; 3 = right kaleidoscope as a guess; or 4 = right kaleidoscope with any level of confidence. Subjects were told that the any-confidence response indicated recognition accompanied by retrieval of specific details about the kaleidoscope or by a vague feeling of familiarity for the kaleidoscope. In other words, any-confidence responses included both remember and know decisions [34]. In contrast, a guess response was made when there was no confidence in the selection and no details were retrieved. Subjects were instructed to make this response when they experienced “no feeling of familiarity for the selected item” when they were “guessing because they were forced to select one kaleidoscope or the other”.

## 3. Results

The proportion of guess responses was high, as shown in Table 1, indicating that subjects found these testing procedures difficult. For trials with one studied kaleidoscope and a similar foil, an average of 56% of the responses were guesses, and the proportion of guessing did not vary according to whether the studied kaleidoscope was shown on the same side as where it was studied [*t*(23) = 0.04]. The level of guessing was somewhat higher than in our previous experiments using central presentation of kaleidoscope images, suggesting that lateral presentation may have made recognition more difficult than in our prior studies. For trials with two new kaleidoscopes, guess responses were also very common and significantly more prevalent than on the trials with a studied kaleidoscope [*t*(23) = 3.17, *p* < 0.01].

**Table 1:** Summary of response data for each presentation condition, showing mean percentage of total responses across the two confidence categories, and response times.

Response confidence	Any-Confidence			Guesses		
Condition	Same Side	Different Side	New-New	Same Side	Different Side	New-New
Percentage of total responses	43.7 (3.6)	43.8 (3.3)	36.7 (3.7)	56.3 (3.6)	56.2 (3.3)	63.3 (3.7)
Response times in ms	1629 (76)	1663 (76)	1717 (86)	1764 (82)	1784 (89)	1778 (101)

Standard error of the mean provided in parentheses.

**Figure 1:**
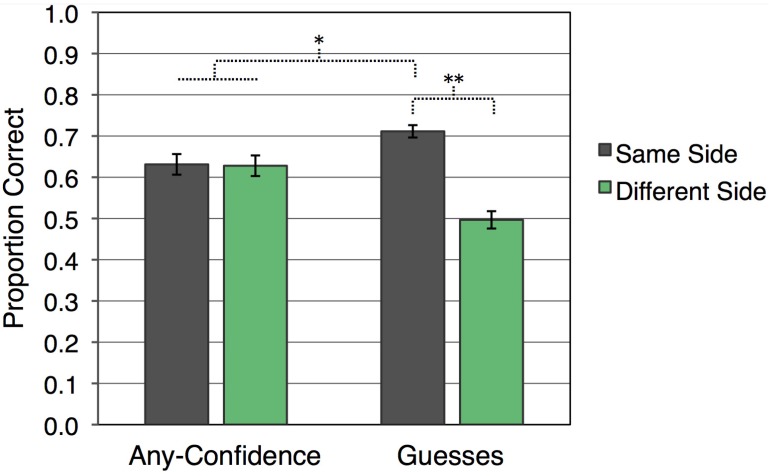
Mean recognition accuracy for each condition (error bars indicate *SEM*; ** *p* < 0.001; * *p* < 0.01).

Recognition accuracy, as shown in Figure 1, was systematically influenced by whether or not there was a match between visual hemifield of presentation at study and at test. There was no overall difference in accuracy for any-confidence compared to guess responses [*F*(1,23) = 1.01]. Yet, recognition responses were more accurate for kaleidoscopes that appeared on the same side during study and test (*M* = 0.672, *SE* = 0.015) than for those that appeared on different sides [*M* = 0.562, *SE* = 0.017; *F*(1,23) = 51.42, *p* < 0.001]. Furthermore, an interaction between response confidence and visual hemifield consistency indicated that this same-side advantage was driven by guess responses [*F*(1,23) = 43.27, *p* < 0.001]. Guess responses were more accurate when kaleidoscopes were presented in the same visual hemifield at study and test (*M* = 0.711, *SE* = 0.015) than when presentations were in different visual hemifields [*M* = 0.497, *SE* = 0.021; *t*(23) = 10.10, *p* < 0.001]. Guessing accuracy for different-side trials was no better than chance [*t*(23) = 0.16], whereas guessing accuracy for same-side trials was significantly above chance [*t*(23) = 13.94, *p* < 0.001]. For any-confidence responses, accuracy was greater than chance for same-side trials [*t*(23) = 5.29, *p* < 0.001] and different-side trials [*t*(23) = 5.03, *p* < 0.001], but accuracy was not reliably influenced by whether kaleidoscopes were presented to the same visual hemifield during study and test [*t*(23) = 0.17]. Accuracy was significantly higher for guess responses with hemifield presentation the same at study and test than for confident responses collapsed across whether or not visual hemifield was consistent [*t*(23) = 3.04, *p* < 0.01]. Additionally, accuracy for confident responses collapsed across hemifield consistency was higher than for guess responses with a different hemifield presentation at study and test [*t*(23) = 4.55, *p* < 0.001].

Response times during the recognition test, as shown in Table 1, were significantly slower for guess responses (*M* = 1775 ms, *SE* = 90) compared to any-confidence responses (*M* = 1669 ms, *SE* = 77) when considering all trial types [*F*(1,23) = 7.28, *p* < 0.05]. There were no response-time differences between new-new trials, trials with an old kaleidoscope that had a consistent hemifield presentation during study and test, and trials with inconsistent presentations [*F*(2,23) = 2.07], and no interaction between type of trial and confidence [*F*(2,23) = 1.09]. Response times in this experiment were generally faster than response times for our previous experiments (e.g., [20]) where subjects were instructed to withhold their responses for 2 s. 

## 4. Discussion

We administered a recognition test devised to reveal an influence of perceptual implicit memory on accurate guessing. Lateralized kaleidoscope images were presented repeatedly during learning under divided-attention conditions. Recognition memory was assessed using a two-alternative forced-choice format. Participants were required to choose between a kaleidoscope stimulus they had viewed 1–2 min earlier and a foil that was extremely similar. 

Under these conditions, guess responses were prominent and reached an average level of accuracy that was above chance and roughly equivalent to that of responses made with some level of confidence. Importantly, kaleidoscope presentation on the same side at study and test produced higher recognition accuracy than when there was a change, but only for guesses. Guessing accuracy for same-side trials reached 71% correct, which is comparable to that in our prior studies, and greater than the accuracy for any-confidence trials. Even though guess responding may not be a perfect way to infer lack of awareness of retrieval on any single trial, the differential results as a function of confidence ratings were indeed informative. An implicit influence of fluency signals was presumably operative on guess trials more than on any-confidence trials, but only when hemifield of presentation at study and test was consistent. Guesses were at chance when visual fluency was disrupted by inconsistent study-test stimulus lateralization. With consistent stimulus lateralization, recognition results clearly deviated from the usual explicit-memory pattern in which guesses are less accurate than responses made with some level of confidence.

The boost in guessing accuracy, when stimulus presentation was in the same visual hemifield during study and test, is consistent with the hypothesis that implicit recognition is dependent on memory storage in contralaterally organized visual cortex. These networks likely mediate repetition-induced changes in fluency, in keeping with current models [1,2,3].

An advantage in accuracy when there was a match of visual hemifield presentation at study and test has also been found in previous studies that used yes/no recognition paradigms [32,35,36]. However, these studies did not separate explicit and implicit memory or monitor response confidence, so it is unclear whether effects were due to implicit memory for the information. Additionally, stimulus meaningfulness has been found to moderate the influence of study-test hemifield consistency, as only recognition for stimuli low in meaningfulness depended on hemifield consistency [35]. Similarly, our kaleidoscope images, which are low in meaningfulness, showed the same visual hemifield advantage, but only for guess responses. Why didn’t participants in our study show this effect for their more confident responses? One speculation is that they sometimes focused on single visual features during learning, and then attempted explicit retrieval of these features during test. This feature-based strategy could have promoted some confidence without much improvement in accuracy, given that many features do not prove useful in distinguishing studied kaleidoscopes from foils. Perceptual fluency for the entire stimulus might be more suitable for this subtle discrimination. Fluency signals may preferentially boost performance when participants do not rely on explicit feature retrieval, paralleling various other examples of decision making without attentive deliberation (e.g., [37]) as well as our previous results suggesting that promoting explicit strategies eliminates the influence of implicit fluency on recognition [22].

Although the present findings are consistent with fluency-based contributions from contralateral visual cortex, it is also possible that other stimulus manipulations at test, such as kaleidoscope size, would show parallel effects (e.g., recognition superior for same-size trials *versus* different-size trials). One way to directly test whether lateralized presentation produces unique effects would be to modify the present design so that stimuli appear above and below fixation instead of left and right of fixation. The present findings on their own do not pinpoint the relevant networks in the brain, and follow-up studies with neuroimaging or magnetoencephalography could be advantageous for directly demonstrating lateralized fluency signals in visual cortex. Such studies could also provide new insights into how visual fluency relates to the neural phenomena of repetition suppression and sensory reactivation.

One of the most remarkable findings from the present experiment is that the highest recognition accuracy was observed for guess responses when side of presentation was consistent. Notably, any-confidence responses could have included high confidence and low confidence responses, and/or both recollection and familiarity. Likewise, our previous demonstration of guess responses with higher accuracy than responses made with any confidence using a paradigm similar to the current experiment (but without lateralized presentations) used an “any confidence” categorization comprised of an uncertain amount of high and low confidence and of recollection and familiarity. However, when recollection and familiarity were indexed by separate responses (remember/know), we found that guess responses were approximately as accurate as remember responses, and that both remember and guess responses were significantly more accurate than familiarity responses [21]. Therefore, the advantage of guess responses over any-confidence responses in the current paradigm was likely not due to an advantage of guessing over highly confident recollection-based responses, but rather to an advantage relative to lower-confidence explicit memory responses (and to the fact that the test was remarkably difficult, likely causing the any-confidence category to be dominated by lower-confidence responses).

The current results bolster the prior evidence summarized above in support of the concept of implicit recognition. Moreover, the advantage for consistent, over inconsistent, side of presentation was here restricted to trials with minimal awareness of memory retrieval. If this advantage does stem from lateralized perceptual fluency signals, then future research should seek to explain why such signals are preferentially available for implicit recognition. Perceptual fluency signals may dominate in particular circumstances, such as when typical effortful retrieval strategies are not engaged. Importantly, the present findings add weight to prior evidence demonstrating recognition outcomes with features that diverge from typical explicit memory findings. When side of presentation was held constant recognition was reliably higher for guess responses that for responses with some level of confidence. A reasonable interpretation is thus that these accurate recognition guesses reflect a preferential engagement of memory mechanisms akin to those responsible for perceptual priming.

## 5. Conclusions

Implicit recognition refers to a memory expression whereby accurate recognition responses are produced without awareness of memory retrieval. We have proposed that mechanisms responsible for perceptual priming underlie implicit recognition in prior experiments. Here, using a divided-visual-field strategy, we obtained evidence consistent with a perceptual fluency account for implicit recognition. When kaleidoscope images were studied in one visual hemifield and then tested in the same visual hemifield, recognition accuracy was higher for guesses than for decisions with any level of confidence, and those guesses were also more accurate than responses made when visual hemifield was inconsistent. Lateralized visual memory storage may be responsible for repetition-induced perceptual fluency that can, under certain conditions, lead to implicit recognition. 
